# The zooarchaeology and isotopic ecology of the Bahamian hutia (*Geocapromys ingrahami*): Evidence for pre-Columbian anthropogenic management

**DOI:** 10.1371/journal.pone.0220284

**Published:** 2019-09-24

**Authors:** Michelle J. LeFebvre, Susan D. deFrance, George D. Kamenov, William F. Keegan, John Krigbaum

**Affiliations:** 1 Florida Museum of Natural History, Gainesville, Florida, United States of America; 2 Department of Anthropology, University of Florida, Gainesville, Florida, United States of America; 3 Department of Geological Sciences, University of Florida, Gainesville, Florida, United States of America; Universita degli Studi di Milano, ITALY

## Abstract

Bahamian hutias (*Geocapromys ingrahami*) are the only endemic terrestrial mammal in The Bahamas and are currently classified as a vulnerable species. Drawing on zooarchaeological and new geochemical datasets, this study investigates human management of Bahamian hutias as cultural practice at indigenous Lucayan settlements in The Bahamas and the Turks & Caicos Islands. In order to determine how hutia diet and distribution together were influenced by Lucayan groups we conducted isotopic analysis on native hutia bone and tooth enamel recovered at the Major’s Landing site on Crooked Island in The Bahamas and introduced hutias from the Palmetto Junction site on Providenciales in the Turks & Caicos Islands. Results indicate that some hutias consumed ^13^C-enriched foods that were either provisioned or available for opportunistic consumption. Strontium isotope ratios for hutia tooth enamel show a narrow range consistent with local origin for all of the archaeological specimens. In contrast, analysis of strontium isotopes in modern Bahamian hutia teeth from animals relocated to Florida from The Bahamas demonstrates that these animals rapidly lost their Bahamian signature and adopted a Florida signature. Therefore, strontium should be used cautiously for determining hutia provenance, particularly for individuals that were translocated between islands. Overall, our findings suggest that ancient human presence did not always result in hutia vulnerability and that the impact to hutia populations was variable across pre-Columbian indigenous settlements.

## Introduction

Across the Caribbean archipelago, zooarchaeological datasets are crucial to documenting the deep history of human impacts on mammalian natural history through time [1–3]. Hutias (Family: Echimyidae; Subfamily: Capromyinae [4]), a group of rodents native to the Greater Antilles, the Cayman Islands, and The Bahamas, are significant for their high rates of endemism, breadth of species and morphological diversification, extreme vulnerability to humans, and broad scale extinction (e.g., >50% extinction rate in the Holocene [5–9]). Of at least 24 documented hutia species, only 11 are living today. This astonishing rate of extinction is readily attributed to a long history of human activities and anthropogenic impacts on hutia habitats and population size [1, 3, 10, 11].

Zooarchaeological records show that during pre-Columbian history (ca. 7,000–500 BP) hutias throughout the region were targeted and consumed as food by indigenous people [12, 13]. Archaeologists and biologists have suggested that some hutia taxa were intentionally managed by pre-Columbian people [12, 14–18] based on evidence such as the intentional translocation of some taxa beyond their native ranges and variable relative abundances of hutia remains across different pre-Columbian sites (e.g., *Geocapromys ingrahami* [14–16]; *Capromys* sp. [19, 20]; *Isolobodon portoricensis* [10, 18]; *Plagiodontia aedium* [10]). Despite ample evidence for hutia exploitation and translocation in the past, empirically identifying ancient hutia management in the Caribbean requires the integration of multiple datasets (e.g., zooarchaeological, geochemical, biochemical) [21].

Here we discuss the scope of indigenous anthropogenic influence over pre-Columbian Bahamian hutia (*Geocapromys ingrahami*) using zooarchaeological and isotopic datasets. From the earliest settlement of the Bahama archipelago (ca. AD 700), Bahamian hutias were a targeted subsistence item among indigenous groups and translocated beyond their native range within the Commonwealth of the Bahamas (The Bahamas) to the Turks & Caicos [12, 14,–16]. At present, the extent of hutia exploitation, translocation, and the timing of hutia extirpation from the majority of the Bahama archipelago is not clear. However, recently radiocarbon dated Bahamian hutia remains from Crooked Island in The Bahamas suggest that some pre-Columbian hutia populations survived initial European incursions at the end of the 15^th^ century and that some continued to persist well into the 17^th^ century (see Table 1 in [22]).

Specifically, this study investigates human management of hutias during the Late Ceramic Age in The Bahamas and the Turks & Caicos Islands. We present the first multi-isotope dataset for pre-Columbian Bahamian hutias. We consider management to be intentional human influence over an animal and/or its environment in such a way that promotes increased abundance and availability for exploitation [21]. In addition to elucidating probable evidence of past human management, these results demonstrate the relevance of zooarchaeological and geochemical datasets to both hutia historical ecology and conservation efforts.

## The Bahamian hutia: Biological and archaeological background

### Diet, population dynamics, and translocation

**Fig 1:**
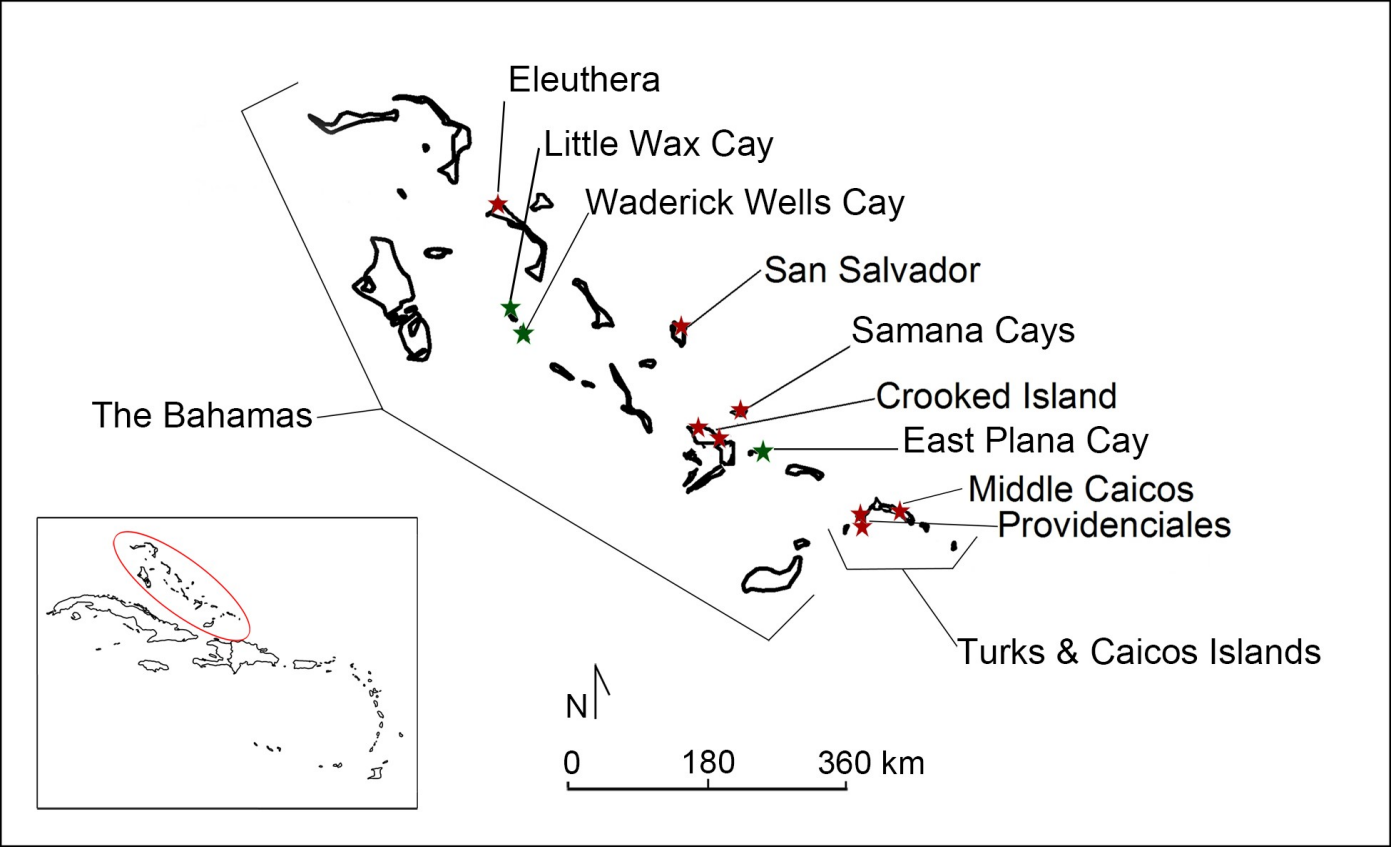
Islands of the Bahama archipelago with archaeological records (red stars) and modern populations (green stars) of Bahamian hutia. Data Source: www.naturalearthdata.com. Prepared by Geoffrey DuChemin.

Collectively, the Bahama archipelago is composed of two politically distinct island groups, The Bahamas and the Turks & Caicos Islands (a British Overseas Territory) (Fig 1). Bahamian hutias are the only endemic terrestrial mammal in The Bahamas and are currently classified as a vulnerable species [23, 24]. Once widespread across the Bahama archipelago, Bahamian hutias are now limited to one ostensibly natural population on East Plana Cay and two modern-introduced populations on Little Wax Cay and Warderick Wells [10] (Fig 1). The three locations with extant populations are not inhabited by humans and conservation efforts are predicated on strict protection from human interference [10]. To date, there is no conclusive paleontological evidence for the natural occurrence of Bahamian hutias among the Turks & Caicos Islands prior to human colonization [24]. This suggests that pre-Columbian archaeological populations of hutias were translocated by humans from The Bahamas to the Turks & Caicos Islands [12].

In 1973, conservation efforts by the Bahamas National Trust lead to the translocation and introduction of six male and five female hutias from East Plana Cay to the hutia-uninhabited island of Little Wax Cay [25]. Based on observations of the wild populations on East Plana Cay and Little Wax Cay [11], Bahamian hutias are considered nocturnal folivores, preferring to forage for food at dusk and inhabiting rocky crevices or silver palm (*Coccothrinax argentata*) leaf piles during the day [14, 26, 27]. Bahamian hutias are known to feed primarily on the ground, are able to absorb sufficient amounts of water through plant consumption [28], and exhibit territorial fidelity (pg. 26–29 in [11]). Bahamian hutias practice a generalized plant foraging strategy, consuming a variety of plant-based food [11]; however, there is evidence suggesting that Bahamian hutias will target particular plants depending on availability (Table 1). Broad dietary breadth has also been observed among captive Bahamian hutias, including the consumption of fruits and vegetables such as carrots (*Daucas carota*) and pears (*Pyrus communis*), as well as chicken and processed dog food [26].

**Table 1:** A list of plants observed or assumed to be preferred among extant Bahamian hutia (*Geocapromys ingrahami*). List of taxa is adapted from Clough [14], Clough and Fulk [2,9], and Jordan [11, 27].

Family	Taxa	Common Name	Reference
Arecaceae	*Pseudophoenix sargentii*	hog-cabbage palm	[11]
Caricaceae	*Carica* sp.	papaya	Table 2 in [14]; [29]
Combretaceae	*Conocarpus erectus*	button mangrove	Table 2 in [14]; [29]
Euphorbiaceae	*Croton lucidus*	firebrush	Table 2 in [14]; [29]
Fabaceae	*Sophora tomentosa*	yellow necklacepod	[11, 27]
Boraginaceae	*Tournefortia gnaphalodes*	soldierbrush	Table 2 in [14]; [29]
Oleaceae	*Forestiera segregata*	Florida privet	Table 2 in [14]; [29]
Phyllanthaceae	*Phyllantus epiphyllanthus*	swordbrush	Table 2 in [14]; [29]
Picrodendraceae	*Picrodendron baccatum*	black ironwood	[11]
Polygonaceae	*Coccoloba uvifera*	seagrape	[11, 27]
Polygonaceae	*Cocoloba diversifolia*	pigeon plum	Table 2 in [11]
Rubiaceae	*Strumpfia maritima*	pride of Big Pine	Table 2 in [14]; [29]
Sapotaceae	*Manilkara jaimiqui*	wild dilly	Table 2 in [11]
Solanaceae	*Solanum bahamense*	canker-berry	[11]

The success of extant Bahamian hutia populations is predicated on a delicate balance of resource availability, the prohibition of human predation or disturbance, and the maintenance of relative ecological and biological stability that can be upset easily via natural events (e.g., hurricanes) or human intrusions (e.g., introduced animals such as dogs or human hunting) [30]. The vulnerability of Bahamian hutias to humans is significant when considering human-hutia interactions in both the present and past. Regarding ease of physical capture, Clough (pg. 810 in [14]) describes their capture by hand on East Plana Cay as “efficient on the beach slope and sand terrace habitats and open shrub thickets on sandy soil, and less effective wherever there were crevices and cave openings in the rock.” Similarly, Jordan (pg. 137 in [11]) states that “given its nearsightedness and waddling gait, *G*. *ingrahami* is easy prey for any half-hearted predator.” As a result, “…wherever predators [including man] are present, hutia are not” (pg. 137 in [11]). However, in a review of captive hutia (Capromyidae) studies, Eisenberg and Woods [31] offer the following observations regarding human-captive Bahamian hutias specifically: 1) reproduction can take place throughout the year when in captivity, 2) juveniles can be independent of parents by two and half months of age, and 3) solid food can be ingested within three days of birth. While at this time there is no known archaeological evidence for Bahamian hutia management via captivity in the past (e.g., features indicative of pen or cage-like structures or preserved concentrations of dung associated with spatial restriction), observations among modern captive populations suggest that the reproductive capabilities of hutias are not immediately hampered by human control over habitat, diet, and socialization.

### The Lucayan archaeological record and Bahamian hutias

Human colonization of the Bahama archipelago began sometime during the 8^th^ century AD by colonists who originated from Cuba, Hispaniola, or possibly both [32, 33]. The indigenous people associated with settlements in The Bahamas are referred to as Lucayan, based upon the name recorded by Spanish explorers during the 15^th^ century [34]. The earliest Lucayan settlements in The Bahamas are recorded on Eleuthera (Preacher’s Cave site, two dates at 2 sigma: cal AD 560 to 720 and ca AD 700 to 980 [35]), and on San Salvador and New Providence (circa AD 800 [31]). In the Turks & Caicos Islands, the earliest settlement documented is on Grand Turk at the Coralie site (cal AD 710 (650 to 885), cal AD 770 (665 to 905) and cal AD 790 (670 to 970) [36]).

The Lucayan settlement pattern is characterized by a large number of closely-spaced small sites with shallow midden accumulations, and an equally large number of special purpose sites (e.g., farmsteads and procurement sites: reef fishing, flats fishing, mollusk collecting, salt collecting, palm nut collecting). Most of the sites are located directly on, or immediately adjacent, to a beach; a location that is annually vulnerable to tropical storms. Recent investigations indicate that many Lucayan settlements were ephemeral and that particular locations were episodically abandoned and reoccupied (e.g., [37]).

The Lucayan economy was based on shifting cultivation in which gardens were cultivated for a few years and then left fallow. The shallow and nutrient-poor soils of The Bahamas will not support more intensive cultivation [38]. Manioc (*Manihot esculenta* Crantz), coontie (*Zamia lucayana* B.), and maize (*Zea mays* L.) starch grains were identified at the Rolling Heads site (LN-101), Long Island. All three were found on a single tiger lucine clamshell scraper (*Codakia orbicularis* L.), along with manioc on a limestone microlith [39]. These cultigens appear to have been cooked together in an adjacent earth oven. The tools and earth oven are from a level that was AMS dated to cal AD 1020–1060 (32.4%) and cal AD 1075–1155 (63%). In addition, Berman and Pearsall [40] report starch grain evidence of maize, chili pepper, and cf. *Xanthosoma* sp. (yautia) or cf. *Zamia* sp. on microliths from the Three Dog site on San Salvador. The simultaneous cultivation of multiple crops, and their processing with a single tool, reflects horticultural production of a suite of cultigens. Although manioc and other “root crops” typically are identified as staples, Veloz Maggiolo and Figueredo each [41, 42] cite ethnohistoric reports as evidence that maize was the most important cultigen in The Bahamas.

Farming was complemented by fishing, mollusk collecting, foraging, and hunting [13, 43–46]. Hutias, iguanas, sea turtles, pond turtles, tortoises, crocodiles, manatees, monk seals, birds, land crabs, and marine crabs have all been identified as probable meat sources, but none of them are ubiquitous, and with the exception of hutias, none have been identified in significant numbers [47, 48].

Zooarchaeological hutia records are documented across the Bahama archipelago, with abundant remains present on some islands but not at all Lucayan settlements. As summarized by LeFebvre et al. [12], reports of hutia remains from archaeological contexts consist of 741 individual Bahamian hutia specimens (individual bones) representing a minimum of 51 individual animals from 13 archaeological sites across The Bahamas and Turks & Caicos Islands. The bulk of published records are from sites on Crooked Island (The Bahamas) and Providenciales (Turks & Caicos Islands) [12, 22] (see Fig 1). Contextually, the vast majority of Bahamian hutia archaeological remains have been recovered from midden deposits and not from archaeological features of known function (e.g., pits, hearths, or ritual deposits).

**Fig 2:**
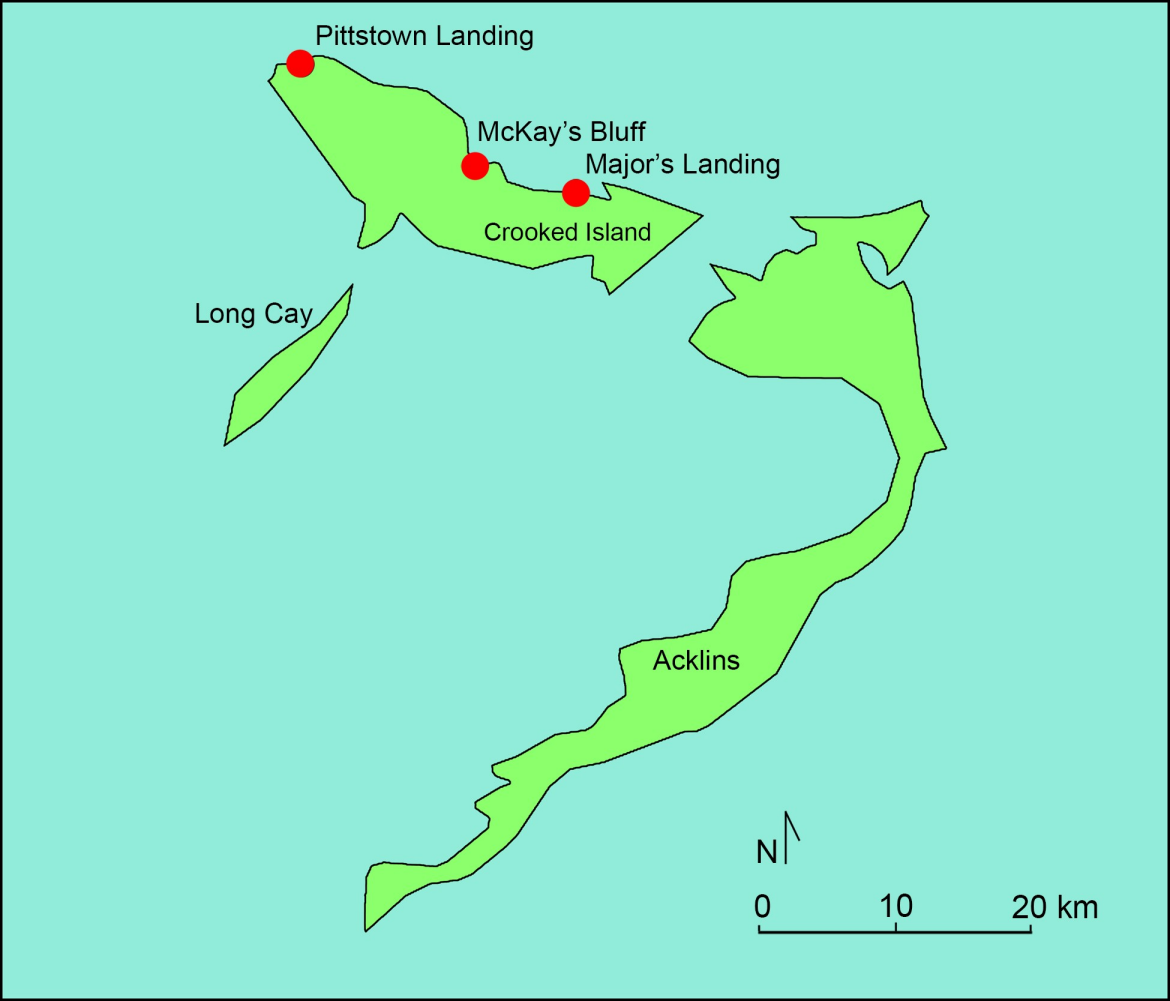
Map showing location of Pittstown Landing, McKay’s Bluff Cave, and Major’s Landing on Crooked Island (The Bahamas). Data Source: www.naturalearthdata.com. Prepared by Geoffrey DuChemin.

**Fig 3:**
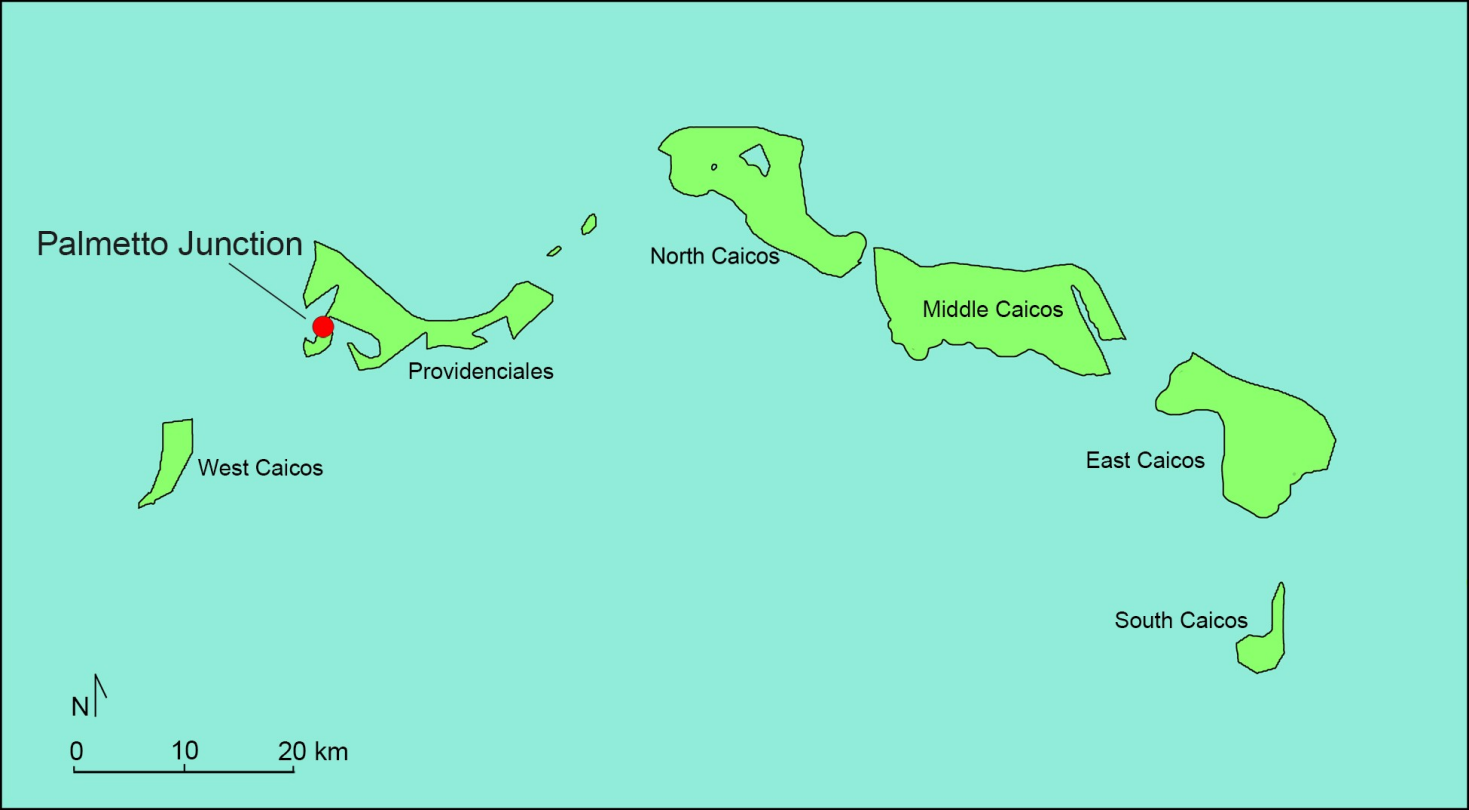
Map showing location of Palmetto Junction on Providenciales, Turks & Caicos Islands. Data Source: www.naturalearthdata.com. Prepared by Geoffrey DuChemin.

Taken together, excavations at three sites on Crooked Island (Major’s Landing (CR-8), Pittstown Landing (CR-14), and McKay’s Bluff Cave (CR-5)) and one on Providenciales (Palmetto Junction) have produced the largest collection of archaeological hutia remains published to date with a total of 686 individual bone specimens reported [12, 22] (Figs 2 and 3). Hutia specimens from Major’s Landing and Palmetto Junction are included in this study. Major’s Landing is located on the north central coast of Crooked Island where excavations yielded 111 individual hutia specimens. Palmetto Junction is located on a narrow isthmus on the western area of Providenciales. Excavations recovered an abundant and well-preserved bone assemblage including the largest known concentration of archaeological Bahamian hutia remains thus far observed in the greater Bahama archipelago. Although analysis is going, thus far 422 individual hutia specimens representing a minimum of 26 individual animals have been reported [12, 15]. [11, 14]. Because Bahamian hutias were not native to the Turks & Caicos Islands, the large hutia assemblage at Palmetto Junction is attributable to human translocation and introduction of the rodent to the island [15]. Both the Major’s Landing and Palmetto Junction vertebrate assemblages were dominated by fish taxa, with hutia supplying a supplementary source of terrestrial-derived dietary protein (e.g., [15]). All hutia specimens at each site were recovered from secure archaeological contexts indicating not only their presence on the islands but also their exploitation and consumption by Lucayan peoples.

Based on direct AMS ^14^C of hutia mandible specimens from Major’s Landing (cal AD 1330–1440) and Palmetto Junction (cal AD 1425 to 1450) [11], both sites date to the Lucayan Period [32]. When considering additional AMS ^14^C dates from an associated crocodile femur at Pittstown Landing (cal AD 1050 to 1250) and hutia specimens at McKay’s Bluff Cave (cal AD 1450 to 1620) [12, 22], it seems that Crooked Island assemblages represent slightly earlier Lucayan occupations and hutia exploitation than on Providenciales, although it is possible that they were at times contemporaneous.

### Recent zooarchaeological analysis

Recent zooarchaeological analysis of hutia remains from Major’s Landing, Pittstown Landing, and Palmetto Junction produced 558 individual cranial, dental, or postcranial specimens with a minimum of 34 individual hutias represented across all three sites [12]. Although the faunal assemblages from these three sites are dominated by fish and shellfish remains [15, 49], the quantity of hutia specimens present indicates that these animals were an important and targeted food source for the people residing at these settlements. Moreover, at Palmetto Junction, not only are the hutia remains more abundant in terms of NISP relative to other archaeological sites, but the individual skeletal elements demonstrate larger body size as well. LeFebvre et al. [12] conducted a morphometric analysis of individual skeletal elements of the Palmetto Junction hutia and compared them to modern hutia captured in The Bahamas. When compared to the modern hutia, several cranial and postcranial elements including the mandibular tooth row, humerus, radius, innominate, femur, and tibia from Palmetto Junction hutia exhibit structures (e.g., greatest breadth of proximal end, greatest breadth of distal end) that are larger in size than the modern comparative specimens. While it is not possible to definitively attribute the increased size of the Palmetto Junction hutias to human influence, the reproductive success and propagation of hutias on Providenciales indicates that these animals had ample food supply and/or possibly received protection from potential wild and domestic sources of predation (e.g., ospreys, boa constrictors, dogs). Provisioning food and providing protection to hutias through cultural practices and human intervention may have contributed to the apparent success and stability of the introduced population at Palmetto Junction [12].

## Methods and materials of analysis

### Isotopic analysis

Isotopic analysis of archaeological remains in the Caribbean has contributed important insights into movement and dietary patterns of human and non-human taxa (e.g., [50–59]). This work benefits directly from the pioneering work of the 1970s and 1980s that demonstrated direct correlation between stable isotope values of foods consumed with the stable isotope values of consumer tissues, both bone collagen [60, 61] and bone apatite [62]. Keegan and DeNiro [63] applied these efforts using stable carbon and nitrogen isotopes derived from bone collagen to demonstrate their value to address diet variability and food web ecology in The Bahamas.

Basic differences in how carbon is assimilated in plants during photosynthesis, namely C_3_ vs. C_4_ pathways, result in non-overlapping carbon isotope ratios (δ^13^C values) for plant producers and consumers that feed on these different kinds of plants. C_3_ plants that include trees, tubers, and most vegetables are ^13^C-depleted relative to ^13^C-enriched C_4_ plants (e.g., maize, amaranth). A third type of photosynthesis that utilizes the Crassalucean Acid Metabolism (CAM) results in plants with intermediate δ^13^C values and includes some succulents like jade plants (Family: Crassulaceae) and cacti (Cactaceae).

Nitrogen isotope ratios (δ^15^N) reflect protein consumed and are particularly useful in assessing relative trophic structure of marine and terrestrial food webs and in distinguishing between terrestrial and marine-based food webs [63]. Collectively, bone collagen studies of animal tissues have demonstrated that δ^13^C and δ^15^N values differentially reflect protein components of food consumption, while bone bioapatite δ^13^C reflects all, or ‘total’ aspects of the diet [64]. This is important because in the Caribbean C_4_ foods as economic resources are less common prior to the introduction/adoption of maize horticulture and it remains unknown how important CAM plants were economically. An important advantage to including bone apatite in studies of food web ecology is to provide a total picture of the diet [64], rather than one biased towards the protein portion of the diet [65]. When bone collagen is well preserved with good yields, δ^13^C values derived from bone apatite tend to be uncompromised and biogenic [62, 64]. Building on these developments, one potentially powerful interpretative tool is to explore the spacing of carbon isotope ratios between the bone apatite (ap) δ^13^C_ap_ and the bone collagen (co) δ^13^C_co_ results from independent assays. This Δ^13^C_ap-co_ value then reflects the extent of particular carbohydrates present in total diet relative to the protein portion of the diet. Recent approaches using this relationship incorporate multivariate tools to explore dietary variation expanding on this complex relationship (e.g., [66–68]).

Light stable isotope ratios of carbon in bone collagen and bone apatite thus serve as proxies of diet in animals, given good preservation of bone/tooth enamel. Heavy radiogenic isotope ratios in contrast, such as strontium (^87^Sr/^86^Sr) include a dietary input, but are more tied to bioavailable strontium that is reflected in geological formations of particular landscapes and is ultimately made available through the plants consumed [69]. Studies of these systems have demonstrated that strontium ratios from tooth enamel are most accurate in reconstructing patterns of variability in faunal remains, as opposed to bone which may be diagenetically compromised if bone collagen has very low yields and poor preservation. In contrast to bone, tooth enamel is resistant to diagenetic changes and tends to preserve the biogenic strontium isotope signal [70]. In particular, tooth enamel from archaeological fauna with small home ranges is a well-established method for determination of the local isotopic range [71, 72].

**Fig 4:**
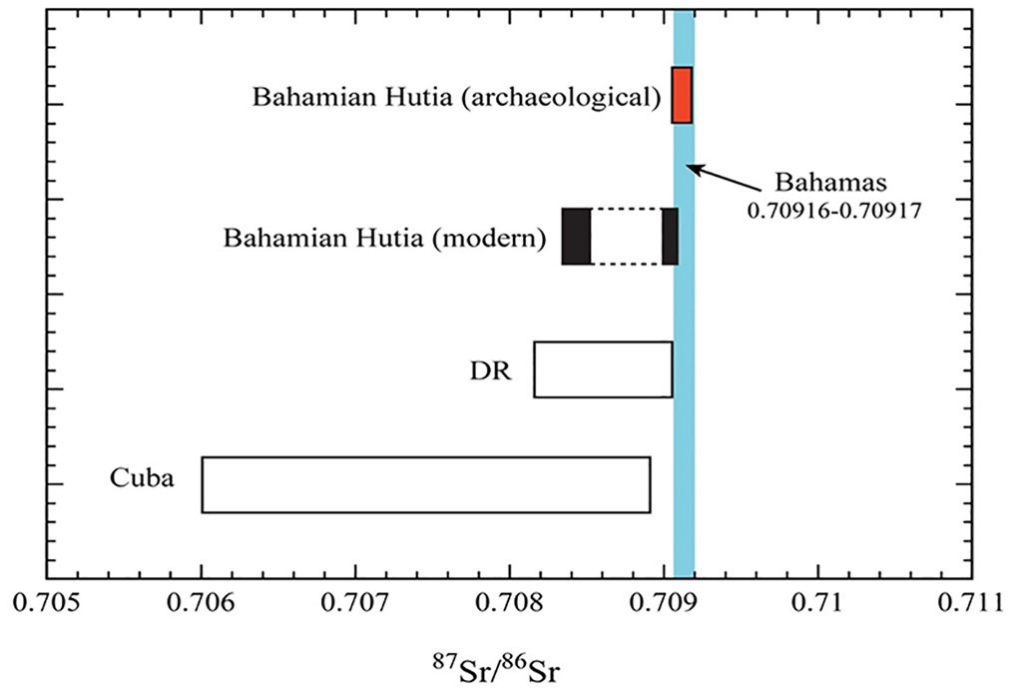
Strontium isotope data for archaeological and modern Bahamian hutias (*Geocapromys ingrahami*) compared to expected strontium isotope variations for the Bahamas, Dominican Republic (‘DR’), and Cuba. Bahamas strontium isotope range based on McArthur and Howarth (2004) [74] seawater curve (see text for details). ‘DR’ and Cuba ranges after Laffoon et al. (2012) [52].

The Bahamas is an extensive archipelago of Quaternary carbonate islands. The oldest exposed formation is around 400,000 years old, formed during marine stage 11 [73]. According to McArthur and Howarth [74], seawater ^87^Sr/^86^Sr will range from 0.709174 to 0.709164 at this time period. As marine carbonates inherit their strontium from the seawater, all carbonate rocks exposed in The Bahamas should have strontium isotopic compositions within this narrow range (0.70917 to 0.70916) (Fig 4). By analogy, all plants and animals from The Bahamas, regardless on which island they lived, should show ^87^Sr/^86^Sr within error of the modern seawater strontium value.

For animals such as hutias, the possibility of incidental consumption of small amounts of dust transported from outside The Bahamas archipelago must be considered. Such dust will be deposited in the local soil and can affect the strontium isotope signal of the plants. Also, the dust can be attached to leaves and other plant parts that may be consumed. The long-range external dust input in The Bahamas is dominated by Saharan dust [75]. Saharan dust ^87^Sr/^86^Sr is mostly between 0.715 and 0.718 [76] and thus much higher (more radiogenic) than any of the seawater precipitated carbonate rocks that dominate The Bahamas geology. However, Schulting et al. [77] conducted an extensive strontium isotope study of modern trees from The Bahamas and the Turks & Caicos Islands and the data were all within the strontium range of modern seawater (see also [78, 79]). Carbonate rocks naturally contain very high Sr concentrations and apparently, the Saharan dust strontium contribution is not significant enough to affect the strontium isotopic compositions of the local soils and plants. As a result, all plants and animals from The Bahamas and Turks & Caicos are expected to be near identical to modern seawater in terms of their strontium isotopic composition [74]. In contrast, Cuba and the Dominican Republic show wider local strontium isotope range [50], with lower ^87^Sr/^86^Sr values compared to The Bahamas (Fig 4). Therefore, animals or plants originating from Cuba and/or Dominican Republic will be easily identifiable as non-local if recovered in archaeological contexts in The Bahamas or Turks & Caicos Islands.

### Analyzed specimens

We conducted isotopic analysis of hutia individuals from Major’s Landing (Crooked Island) and Palmetto Junction (Providenciales) to characterize individual patterns of food consumption and animal provenance. Well-preserved archaeological hutia mandibles with molars were selected because they are taxon-specific and tooth/bone from the same individual could be assayed for multiple isotope ratios. Sampling mandibles with dentition also ensured that we were not unintentionally sampling the same individuals. Second molars were specifically selected to document a post-weaning signal that could be compared across individuals in the zooarchaeological sample. In total, nine specimens representing nine individual hutias from archaeological contexts were assayed from Palmetto Junction (n = 7) and Major’s Landing (n = 2). All archaeological hutia specimens are curated and accessible at the Florida Museum of Natural History (FLMNH) in Gainesville, FL USA. The hutia specimens from the Major’s Landing site on Crooked Island are curated in the Environmental Archaeology Laboratory under Accession #396 with permission to study granted by Associate Curator Kitty Emery. The hutia specimens from the Palmetto Junction site on Providenciales are curated in Caribbean Archaeology under Accession # 2013–40 with permission to study granted by Curator William Keegan.

In addition, four modern Bahamian hutia (FLMNH specimen numbers: 22383, 22385, 23208, 23209) were included for comparative purposes, sampled from the Mammalogy collections at the FLMNH. Permission to study was granted by Curator David Reed. These modern samples were collected from East Plana Cay, however, two specimens represent individuals that were translocated live to Gainesville, Florida for captive study at the FLMNH.

Mechanical and chemical pretreatment of all bone and tooth enamel samples was done in the Bone Chemistry Lab, Department of Anthropology, University of Florida. Second molar (M_2_) tooth enamel was separated from associated dentine mechanically with the aid of a Leica stereo microscope and a mounted NSK dental drill with a Brassler tungsten carbide bit. Individual tooth enamel samples were subsequently re-inspected under light microscopy and any dentine-associated fragments were removed prior to reduction and further analysis. In total, ca. 20–30 mg of cleaned tooth enamel pieces were obtained. For mandibular bone, a small sample (< 0.5 g) was cut from each individual mandible and reserved for light isotope analysis.

Cleaned tooth enamel was analyzed for Sr ratios (^87^Sr/^86^Sr) and stable isotopes of oxygen (δ^18^O_en_) and carbon (δ^13^C_en_) to deduce geographical origin and characterize individual diet. Sampled bone was analyzed for stable isotopes using both the bone mineral, or bioapatite, fraction (δ^18^O_ap_ and δ^13^C_ap_) and bone protein, or collagen, fraction (δ^15^N_co_ and δ^13^C_co_) to further elucidate individual dietary pattern. All mass spectrometry was conducted in the Department of Geological Sciences, University of Florida.

For stable isotopes, ca. 8–10 mg of cleaned tooth enamel was reduced using an agate mortar and pestle, weighed, and placed in a weighed 1.5 mL microcentrifuge tube. Although tooth enamel is 98% inorganic, each sample was oxidized for 8 hrs at room temperature with a 50:50 solution of double deionized, distilled water (DI-H_2_O) and sodium hypochlorite (NaOCl) to remove potential organics, and rinsed to neutral pH with ultrapure H_2_O. To remove potential secondary carbonates, a 0.1 M acetic acid (CH_3_COOH) solution was added for an additional 8 hrs, and samples were then rinsed to neutral pH with ultrapure H_2_O, frozen and then lyophilized (freeze-dried) for ~48 hrs.

Sampled bone was reduced using a ceramic mortar and pestle and separated into two fractions for light isotope analysis. For bone bioapatite, ca. 20–25 mg of the smaller bone fraction (< 0.25 mm) was weighed and placed in a 1.5 mL microcentrifuge tube. All subsequent chemical pretreatment procedures followed tooth enamel methods outlined above, although bone samples were oxidized for ~16 hrs. For bone collagen, ca. 250 mg of (0.25–0.5 mm) was placed in a 15 mL centrifuge tube, diluted with ca. 12 mL of 0.5 M HCl at room temperature, refreshed every 24 hrs, for five days, rinsed to neutral pH, and ca. 12 mL of 0.125 M sodium hydroxide (NaOH) was added to remove exogenous humates. Samples were then transferred to 20 mL scintillation vials with ca. 10 mL of 10^−3^ M HCl and heated at 95°C for 5 hrs, spiked with 10 μL 1 M HCL, and heated at 95°C for another 5 hrs. Samples were then removed, centrifuged, and the purified collagen solution was reduced to ca. 2 mL at 60°C, frozen, and lyophilized for ~72 hrs.

Strontium separation from all sampled tooth enamel was conducted in a class 500 clean lab, equipped with class 100 laminar flow hoods. Cleaned tooth enamel pieces were weighed and dissolved in pre-cleaned Teflon vials by heating for 24 hrs in 8N optima-grade nitric acid (HNO_3_). Sample vials were opened and evaporated to dryness in a laminar flow hood. The dried residues were dissolved in 3.5N HNO_3_ and Sr was separated through ion chromatography using a strontium-spec resin (Eichrom Technologies, Inc.) and collected with 4x distilled H_2_O.

For structural carbonates (tooth enamel and bone bioapatite), samples were loaded into glass vials and placed into a Kiel III carbonate preparation device, reacted with a 100% orthophosphoric acid at 70°C. Resulting sample CO_2_ passed through a ConFlo IV preparation system paired with a Finnigan MAT 252 IRMS. All δ^18^O and δ^13^C values measured are reported in standard delta (δ) notation relative to Vienna Pee Dee Belemnite (VPDB) following the standard formula: δX = ([R_sample_/R_standard_]– 1) 1000 where X is the ratio of heavy to light isotope (i.e., ^13^C/^12^C, ^18^O/^16^O). Analytical precision was 0.011 for δ^13^C and 0.038 for δ^18^O based on analysis of NBS-19 standards (n = 8). For bone collagen, samples were loaded into tin capsules and placed into an automated zero blank carousel on a Carlo Erba NA1500 CNHS elemental analyzer to determine C:N, and then transported via He into a Thermo Finnigan DeltaV IRMS via a ConFlo III preparation system. Analytical precision was 0.193 for δ^13^C and 0.092 for d^15^N’ based on analysis of the USGS 40 standards (n = 5). Bone collagen atomic C:N ratio and % nitrogen and % carbon yields were used as proxies for potential diagenesis.

Strontium ratios were measured on a “Nu-Plasma” multiple-collector inductively-coupled-plasma mass spectrometer (MC-ICP-MS). Results are reported relative to NBS 987 ^87^Sr/^86^Sr value of 0.71024 (±0.00003 2σ). All isotope results generated in this study are presented in Table 2.

**Table 2:** Bahamian hutia (*Geocapromys ingrahami*) isotope data from lower second molar (M2) tooth enamel and associated mandibular bone. Modern hutia samples have been corrected for the Suess Effect by adding 1.5‰ to the original δ^13^C value [80].

Site	Provenience	BCL #	^87^Sr/^86^Sr	δ^13^C_en_^1^	δ^18^O_en_	δ^13^C_ap_^1^	δ^18^O_ap_	δ^15^N_co_	δ^13^C_co_^1^	Δ^13^C_ap-co_	wt %N	wt %C	C:N	Bone % C_4_^2^	Enamel % C_4_^2^
				(‰, vs VPDB)	(‰, vs VPDB)	(‰, vs VPDB)	(‰, vs VPDB)	(‰, vs AIR)	(‰, vs VPDB)	(‰, vs VPDB)					
Palmetto Junction	Unit A, Level 3	3848	0.709174	-9.1	-0.9	-6.9	-2.9	6.4	-19.4	12.5	10.58	29.54	3.3	67.2	51.5
Palmetto Junction	Unit A, Level 3	3849	0.709160	-8.7	-1.4	-9.7	-0.9	6.0	-19.6	9.9	14.50	39.65	3.2	47.1	54.4
Palmetto Junction	Unit A, Level 7	3850	0.709166	-8.6	-0.5	-6.6	-3.0	5.4	-19.7	13.1	10.27	28.95	3.3	69.6	54.9
Palmetto Junction	Unit C, Level 3	3851	0.709138	-8.3	-1.0	-10.9	-0.7	6.1	-18.7	7.8	13.37	36.78	3.2	38.7	57.4
Palmetto Junction	Unit C, Level 4	3852	0.709204	-9.8	-1.1	-7.0	-2.3	6.6	-20.0	13.0	13.83	37.98	3.2	66.4	46.4
Palmetto Junction	Unit C, Level 3	2809	0.709162	-11.6	-1.2	-12.4	-1.3	5.5	-19.5	7.0	13.11	36.62	3.3	27.8	33.5
Palmetto Junction	Unit A, Level 3	2810	0.709174	-11.4	-0.9	-7.9	-2.5	5.3	-19.7	11.8	12.68	36.03	3.3	60.0	34.8
Major’s Landing	TP1, Level 5	2811	0.709133	-13.4	-2.9	-12.1	-2.0	3.4	-20.2	8.1	13.66	38.92	3.3	30.0	20.5
Major’s Landing	TP1, Level 5	2812	0.709163	-12.1	-1.6	-11.4	-2.3	4.7	-20.0	8.6	12.54	36.63	3.4	35.0	30.2
Modern Bahamas	FLMNH 22383 ‘Junior’		0.708478	-12.4	-3.7	-13.3	-1.8	7.4	-17.9	4.6	14.06	39.14	3.2	13.1	27.9
Modern Bahamas	FLMNH 22385 ‘Ron’		0.708271	-12.5	-2.4	-12.3	-1.2	7.9	-17.7	5.4	14.31	39.79	3.2	19.4	27.1
Modern Bahamas	FLMNH 23208		0.709143	-11.1	0.3	-11.1	2.7	8.2	-16.8	5.7	13.12	36.46	3.2	26.9	37.1
Modern Bahamas	FLMNH 23209		0.709147	-11.0	0.8	-11.2	1.0	9.7	-17.3	6.1	13.80	37.42	3.2	26.3	37.9

Subscript ‘en’ refers to tooth enamel apatite, ‘ap’ refers to bone apatite, and ‘co’ refers to bone collagen. % C_4_ was determined using -26‰ as the endpoint for 100% C_3_, -12‰ for 100% C_4_, and 9.7‰ for Δ^13^C_ap-co_ spacing in the formula %C_4_ = (-26-(δ^13^C_ap_ -9.7)/-16)*100 (adapted from Somerville et al. [68]).

## Results

### Bahamian hutia translocation

**Fig 5:**
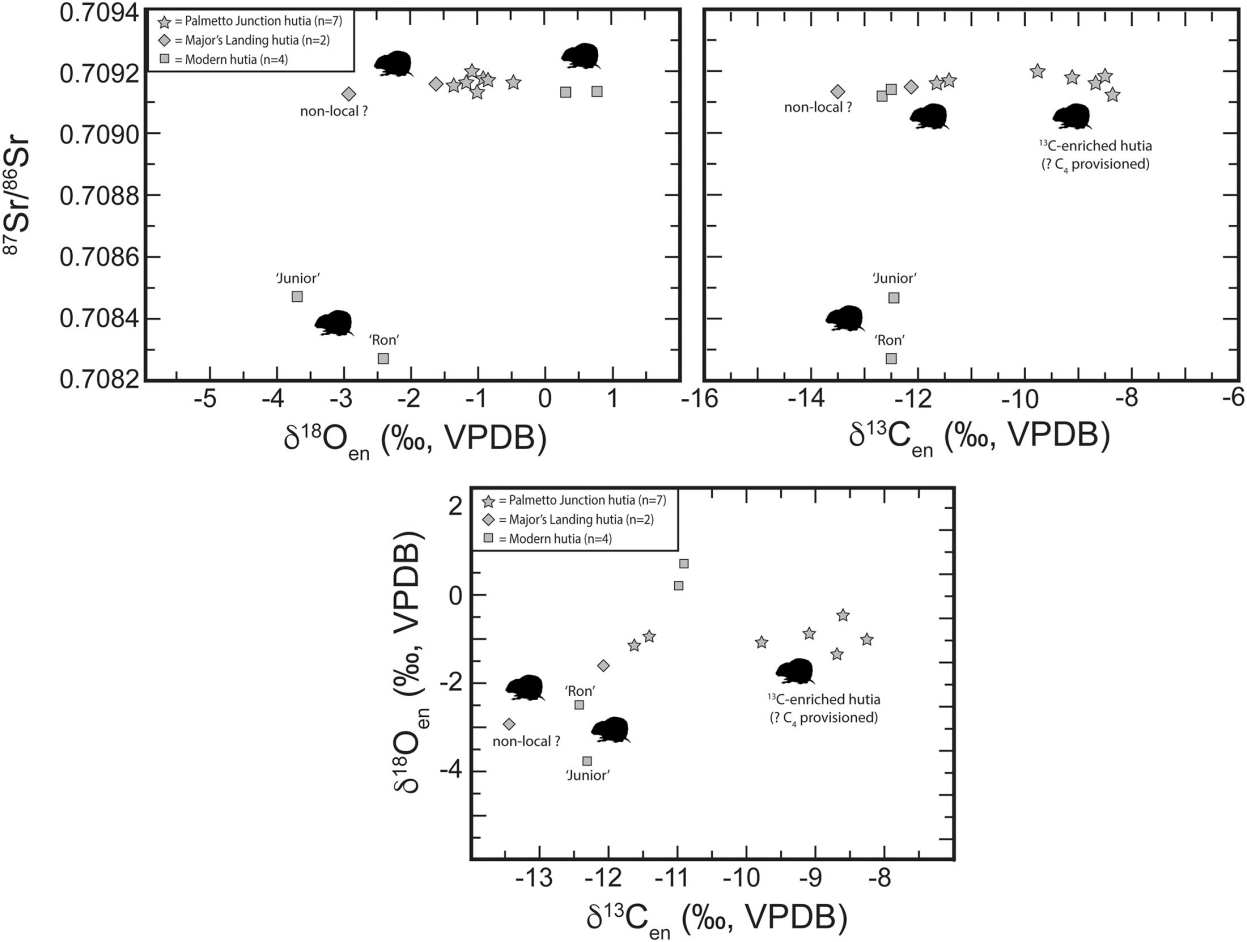
Tooth enamel isotope data for Bahamian hutias (*Geocapromys ingrahami*) analyzed in this study. 5a) (top two boxes) Bivariate plot of δ^18^O and ^87^Sr/^86^Sr and δ^13^C and ^87^Sr/^86^Sr. 5b) (bottom box) Bivariate plot of δ^13^C and δ^18^O. Note distinct radiogenic strontium isotope values and lower δ^18^O for the two modern hutias, ‘Junior’ (FLMNH 22383) and ‘Ron’ (FLMNH 22385), translocated to Gainesville, Florida. The ‘non-local?’ Crooked Island individual sampled (BCL# 2811) exhibits lower δ^18^O and δ^13^C values, which suggests it was subject to a different feeding regime compared to other zooarchaeological Bahamian hutias sampled.

On Figs 4 and 5a, strontium ratios of hutias are compared to The Bahamas and Cuban and Dominican Republic geological baselines. Zooarchaeological hutia sampled from Palmetto Junction and Major’s Island show a very narrow range, between 0.70913 and 0.70920. This range falls within the error of strontium isotope ratios of modern-day seawater (^87^Sr/^86^Sr = 0.70917) [69] based on the reported reproducibility for the NBS 987 standard (±0.00003 2σ) analyzed together with the samples. All of the archaeological hutia specimens thus exhibit ^87^Sr/^86^Sr within the range of carbonate rocks from The Bahamas, indicating local origin.

The four modern Bahamian hutia enamel samples show ^87^Sr/^86^Sr values from 0.70827 to 0.70915. Therefore, two of the modern Bahamian hutia specimens show strontium isotopes that are much lower than expected for the Bahamas. These two individuals, named ‘Ron’ and ‘Junior’, were born in The Bahamas and transported to Gainesville, Florida in the early 1980s. Ron arrived in Gainesville on August 10, 1983 and Junior arrived March 25, 1981. Both individuals lived and died in captivity at a research lab in Gainesville managed by the FLMNH. Ron lived for just under three years prior to his death and Junior for about five and half years (data on file at the Division of Mammals, FLMNH). Drinking water in Gainesville, Florida has strontium values around 0.7079, well beyond the range expected for The Bahamas. Hutia teeth, including their tooth enamel, constantly regenerate through life [81]. While the timing of tooth growth, wear, and regeneration among Bahamian hutias is not known, our data indicate that the two modern individuals lived long enough in Florida to equilibrate with the local Sr isotope range. Significantly, if archaeological hutias were introduced to new locales, they may rapidly (i.e., <3 years) adopt the local signature and obscure their origins; therefore, within archaeological and modern studies of animal mobility and provenance caution should be used in interpreting strontium results from tooth enamel when used to determine the origins of animals that have rapidly or continually growing teeth, such as rodents. Nevertheless, if archaeological animal remains in The Bahamas show lower or higher strontium values than the expected narrow range for the Bahamian archipelago, then this would indicate that the animal was translocated and died before the teeth equilibrated with the local environment. Therefore, strontium isotopes can be useful for identification of archaeological remains in cases where animals were translocated and subsequently died rapidly, before their teeth equilibrated with the new environment. Furthermore, strontium isotopes can be useful for tracking of imported animals from outside The Bahamas, if the remains exhibit distinct strontium signatures from the expected local ^87^Sr/^86^Sr range.

### Bahamian hutia diet

For δ^18^O_en_ (Fig 5), the two modern hutias moved to Florida are ^18^O depleted as is one of the two individuals sampled from Major’s Landing on Crooked Island (BCL# 2811). The one individual from Crooked Island with lower δ^18^O_en_ also exhibits a lower δ^13^C_en_, but has a strontium signature consistent with local values. This individual may have obtained its drinking water/food from a different source from the other individual sampled from this site, or it may be that it is non-local. The other two modern hutias from East Playa Cay are ^18^O-enriched, and both bracket the observed δ^18^O_en_ variation in zooarchaeological hutias. Due to complexities of hutia physiology and body water δ^18^O inputs and outputs, however, variation observed may be from provisioned water sources for the modern hutia.

For δ^13^C_en_ (Fig 5), five hutias from Palmetto Junction exhibit higher δ^13^C which suggests potential C_4_ input, or marine-based foodstuffs that would be less likely given the feeding regimes observed for hutia as obligate vegetarians. As described above, archaeological and ethnohistoric sources indicate Lucayan groups cultivated maize, a C_4_ plant. Generally, while photosynthesis pathways (e.g., C_3_, C_4_, CAM) are taxon specific, there is no accepted taxonomic δ^13^C value for any given plant, rather there are ranges of δ^13^C values based on micro and macro environmental conditions (e.g., insolation, drainage, tree cover) across a given landscape. At present, there is no comprehensive listing or body of scholarship discussing identifications of either CAM or C_4_ plants for The Bahamas, making it difficult to reconcile dietary isotopic signatures with potentially consumed plants. For example, among the preferred plant taxa consumed by extant hutias listed in Table 1, none are currently designated as CAM plants. However, two families represented in Table 1, Rubiaceae and Euphorbiaceae, include some CAM species; therefore, we cannot at this time definitively rule out that some hutias may have consumed native CAM-based plants. Moreover, all other hutias, including all modern hutia sampled, are depleted in ^13^C with lower δ^13^C values consistent with the C_3_-based food webs constructed by Keegan and DeNiro [63] and more recent publications (e.g., [65]). Moving forward, field sampling and isotopic analysis of both modern and archaeological plant specimens will continue to refine models and help improve resolution of multi-scalar comparative baselines across variable environmental conditions.

**Fig 6:**
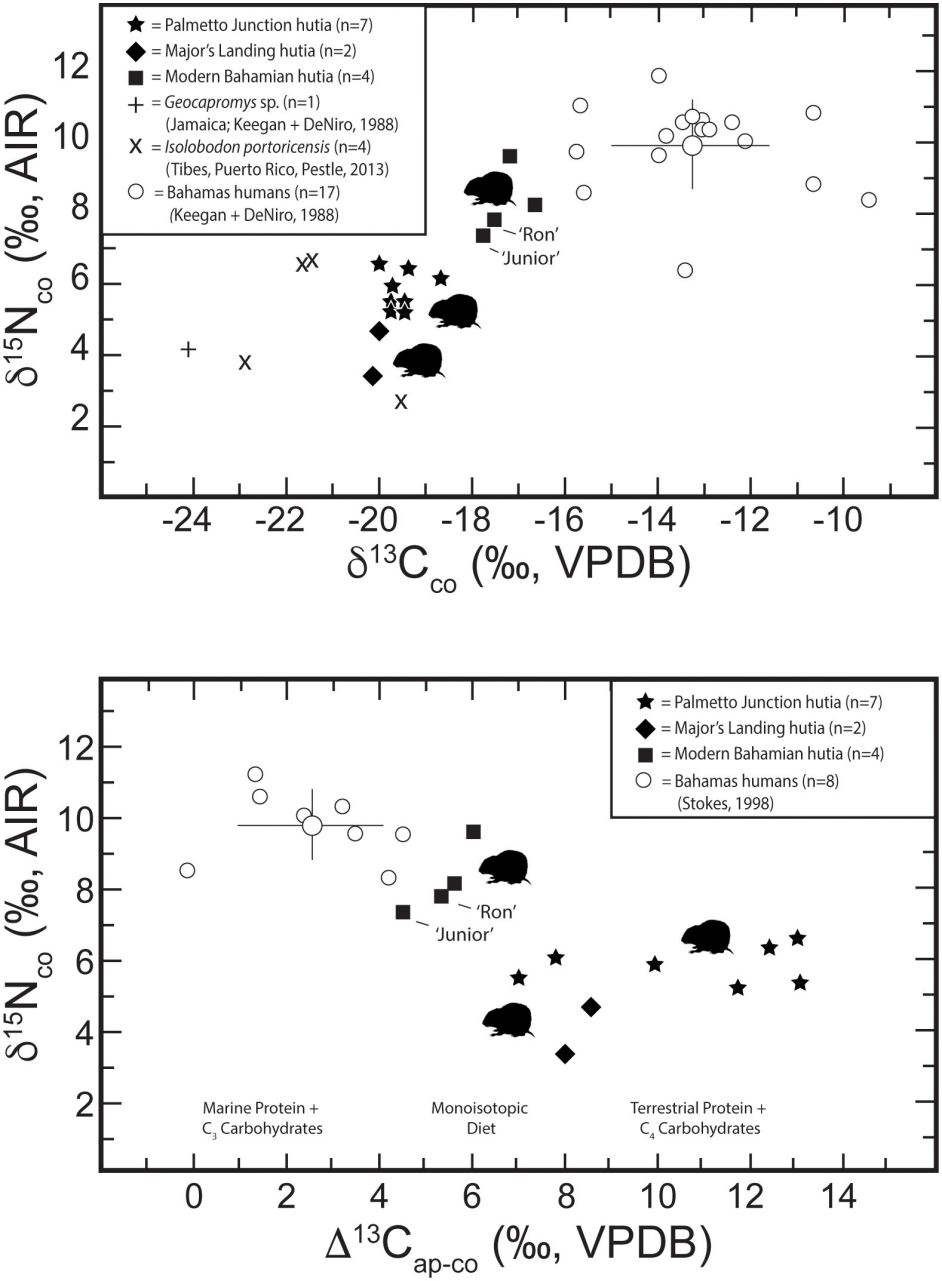
Bone collagen and bone apatite isotope data for Bahamian hutias (*Geocapromys ingrahami*) compared to data from published sources. 6a) (top box) Bivariate plot of δ^13^C and δ^15^N from bone collagen. Note ^15^N-enriched human sample with elevated δ^15^N reflecting consumption of significant marine-based protein compared to vegetarian hutia. Note also the ^13^C-depleted *Geocapromys* individual from Jamaica, which likely represents 100% terrestrial C_3_ carbohydrates. 6b) (bottom box) Bivariate plot of carbon ‘spacing’ (Δ^13^C_ap-co_) and δ^15^N from bone collagen. With respect to carbon ‘spacing’, the human isotope data reflects principally marine-based protein and C_3_-based terrestrial carbohydrates. ^13^C-enriched hutia with Δ^13^C_ap-co_ values >9‰ exhibit δ^13^C_ap_ values that suggest a diet provisioned, in part, by C_4_ carbohydrates (see Table 2 for estimated % C_4_ for bone apatite and tooth enamel).

We also analyzed bone collagen and bone apatite from associated jaw bone for each hutia individual sampled from Major’s Landing and Palmetto Junction. For bone collagen (δ^13^C_co_ and δ^15^N) and bone apatite (δ^13^C_ap_), Bahamian hutia results are plotted in Fig 6 and compared with prehistoric human data from The Bahamas [63] as well as related species data from the region, including one *Geocapromys* sp. from Jamaica [63] and for *Isolobodon portoricensis* from Tibes, Puerto Rico [56]. Mean bone collagen hutia values for δ^13^C = -19.5‰ and δ^15^N = 5.9‰ broadly support a mixed C_3_-based dietary regime and are consistent with hutia data reported from McKay’s Bluff Cave [22]. Compared to human remains sampled for paleodietary analysis [58, 63], these hutia data are consistent with a vegetarian-based pattern of food consumption. However, in contrast to the pre-Columbian human results (and the modern hutia results), four hutias from Palmetto Junction exhibit large Δ^13^C_ap-co_ values (> 11‰) that supports a C_4_-based carbohydrate food source in addition to C_3_-based protein. These data from bone are lifetime averages of dietary intake, the bone collagen representing the protein component of bone, while the bone apatite representing ‘total’ diet. Interestingly, the tooth enamel δ^13^C and bone apatite δ^13^C values are disparate which is likely a result of differential rates of carbon assimilation and/or turnover for these two tissues. Further research is required to explore this complex relationship.

**Fig 7:**
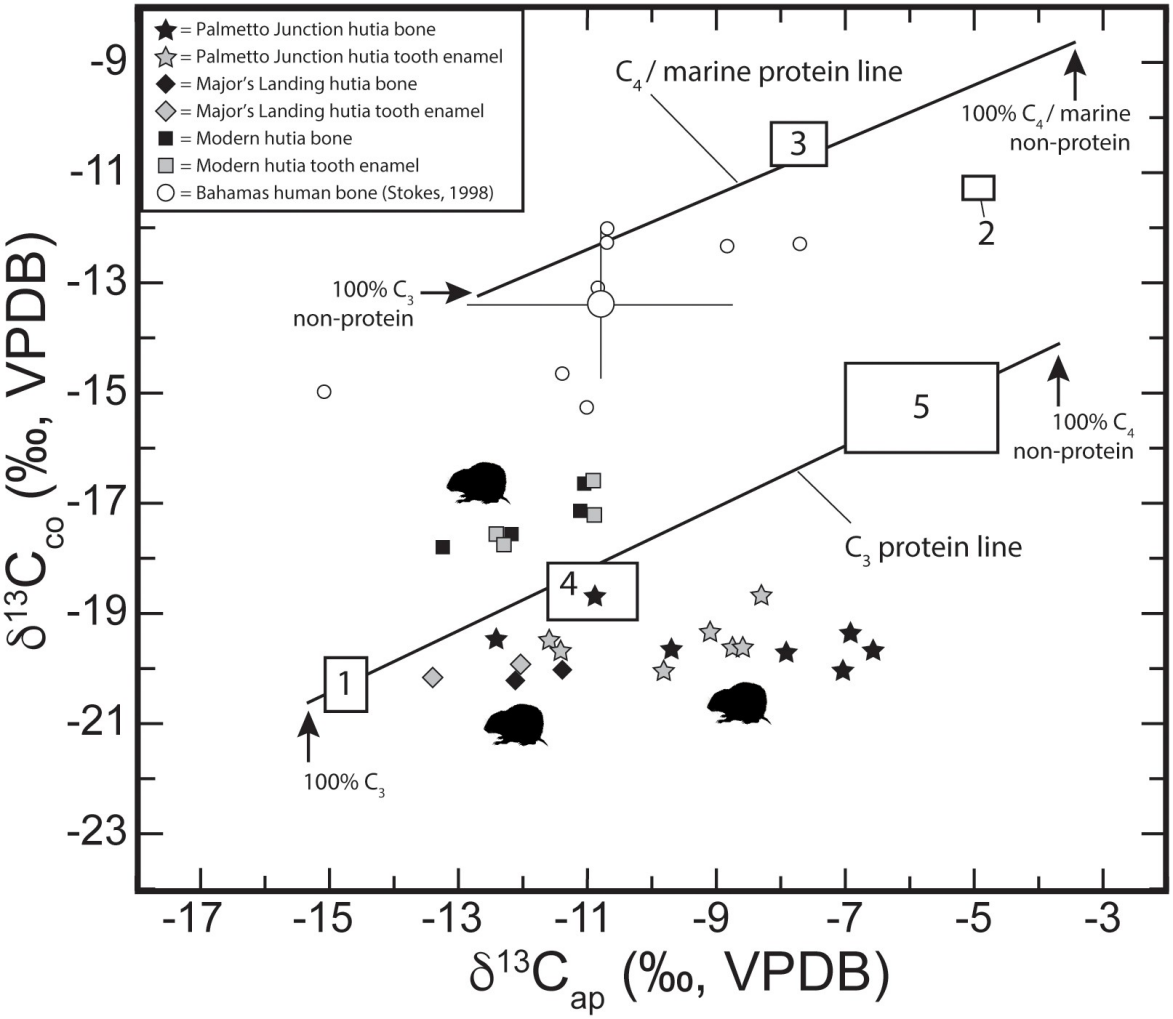
Bivariate plot of carbon bone apatite and carbon bone collagen values (δ^13^C_ap_ and δ^13^C_co_) for prehistoric and modern hutias sampled in this study. Prehistoric human isotope data plotted for comparison [58] include mean and standard error (1σ). C_3_ and C_4_ ‘protein lines’ are based on controlled diet studies of rats [82] and identified to dietary clusters 1–5 using k means cluster analysis [67]. Cluster number is placed at approximate location of ‘mean’ value and boxed area represents standard error (1σ); except for cluster 4, which is displaced by the star for a Palmetto Junction individual.

**Fig 8:**
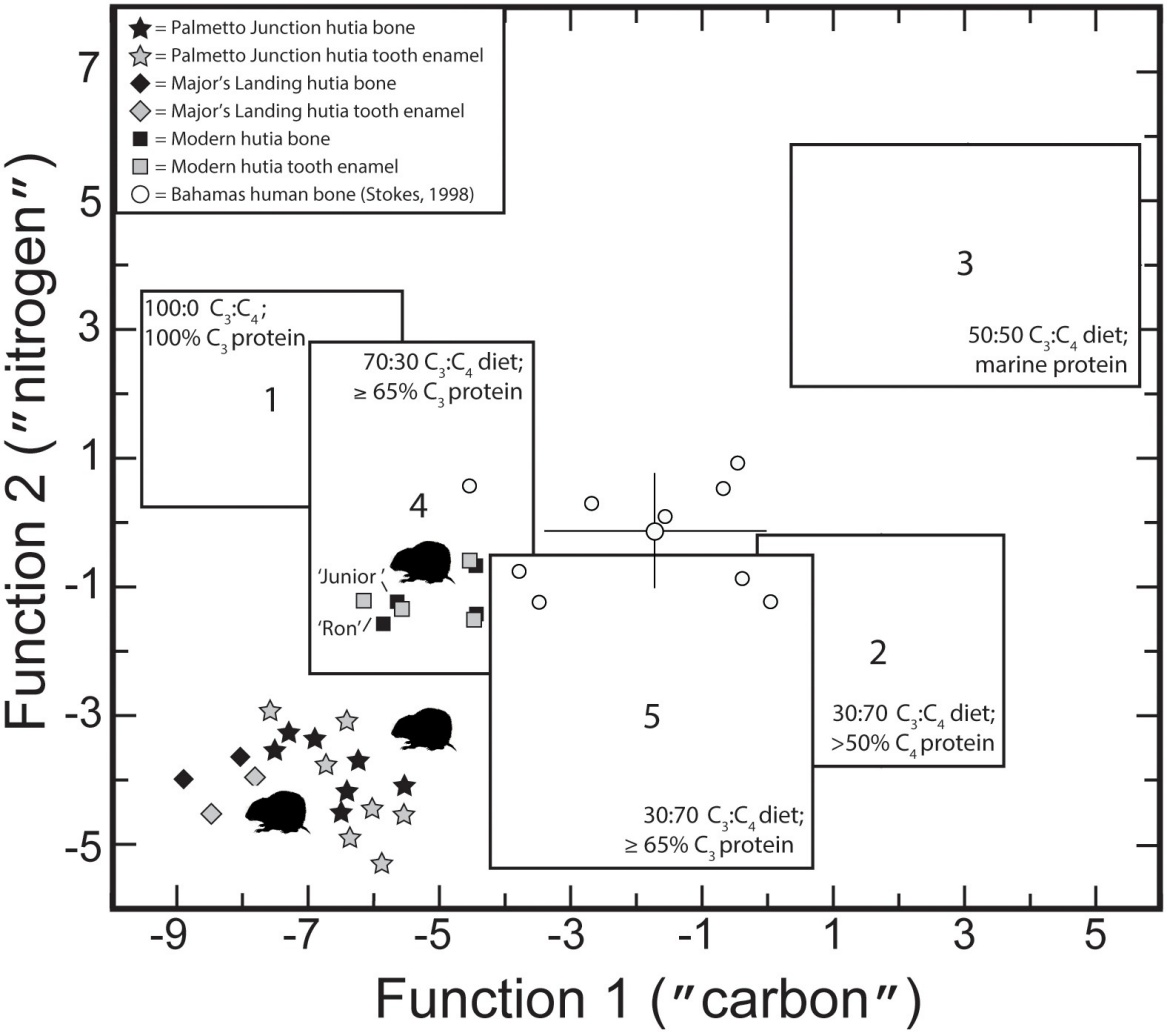
Bivariate plot of discriminant function values for five identified rodent diet clusters based on reanalysis of controlled feeding studies [67] and Bahamian hutia analyzed in this study. Note all zooarchaeological hutia specimens fall outside clusters identified for controlled diet feeding studies, suggesting a different and potentially unique dietary profile for prehistoric Bahamian hutias.

Building on these relationships, we adopt the approach presented by Froehle and colleagues [66, 67] exploring the relationship between carbon from bone collagen and bone apatite. Fig 7 presents a bivariate plot of hutia sampled in this study compared to prehistoric human data from Stokes [58], including mean δ^13^C_co_ and δ^13^C_ap_ and standard error (1 sigma). Here, C_3_ and C_4_ marine protein lines derive from controlled feeding studies of rats (*Rattus norvegicus domesticus*) fed seven different types of purified diet [82]. Importantly, these data and the hutia data reported in this study are not taxonomically driven, per se, but rather a reflection of consumer behavior during the lifetime of each individual hutia sampled. The modern controlled dietary regimes for rats [82] provide a comparative basis for broadly identifying and characterizing dietary differences based on proportions of C_3_ or C_4_-type foods in the diet and these data can be interpreted across taxa, including hutias sampled in this study. For example, the pre-Columbian hutia data for bone apatite and tooth enamel apatite all fall below the C_3_ protein line, but several hutias trend towards cluster 5 (which included rodents fed a 70% C_4_ diet and 65% C_3_ protein; see Fig 8). In contrast, the pre-Columbian human data fall below the C_4_ / marine protein line and are consistent for marine-based protein consumption. However, interestingly, two human individuals do lean towards cluster 2 (which included rodents fed a 70% C_4_ diet and 50% C_3_ protein).

Fig 8 presents these same data using discriminant functions to characterize the controlled feeding studies of rats along ‘carbon’ and ‘nitrogen’ space [67]. All pre-Columbian hutias sampled in this study fall outside of ‘known’ diets based on the modern rat studies. These prehistoric hutias seem to reflect a completely unique diet that has not been characterized to date. What accounts for this pattern is the extremely low ‘nitrogen’ that seems ubiquitous in a reef-based marine/terrestrial system [63] and the more varied ‘carbon’ with the identified provisioned individuals falling to the right of the cluster. As mentioned above, the light isotopes from tooth enamel also exhibit an interesting pattern, with elevated δ^13^C values > -10‰ in five individuals, that suggests a ^13^C-enriched dietary source that may well include a C_4_ component. This seems the likeliest scenario; however, it would be interesting to see results of CAM plants in controlled feeding studies to infer their role in ‘carbon’ discriminate function space.

## Discussion

### Bahamian hutia management

The arrival of humans to The Bahamas ca. AD 700/800 undoubtedly had adverse impacts on hutia populations through hunting and landscape changes. Contemporaneous with human settlement, horticultural activities would have created habitats conducive to hutia population growth as well as the growth of individual animals over time via access to additional food resources (see [12]). Horticultural landscapes would have facilitated garden hunting strategies, including hutia capture by hand, trap, and/or possibly aided by dog. Although we do not know the exact timing of human-hutia interactions after Lucayan colonization of The Bahamas, the zooarchaeological data along with the isotopic data from Major’s Landing and Palmetto Junction indicate that some hutia populations were accessing human landscapes and were available for human exploitation well into the 14^th^ and 15^th^ centuries. We suggest that at some Lucayan settlements a symbiotic relationship between indigenous peoples and hutias may have initially developed around horticultural activities (e.g., garden hunting), perhaps first in The Bahamas, and then human-hutia interaction followed a trajectory toward the more purposeful manipulation and management indicated at Palmetto Junction (see also [12]). Nevertheless, it remains unclear what the population balance between “wild” and those under human “care” might have looked like through time and across different islands.

Contemporary studies of the hutia population introduced on Little Wax Cay provide a basis for elucidating Bahamian hutia historical ecology in the past [11, 27]. Fifteen years after the initial introduction of eleven hutias to Little Wax Cay, Jordan [11, 27] reported an estimate of 1265 hutias present on the island in 1985, concluding that hutias were thriving on Little Wax Cay over that fifteen-year time span. However, the establishment of the hutia colony was not without consequence to the island’s vegetation. Jordan’s survey of the vascular plants across the island indicated a significant reduction in plant biodiversity due to hutia foraging, including the apparent eradication of some taxa (e.g., seagrapes (*Coccoloba uvifera*), hog-cabbage palm (*Pseudophoenix sargentii)*, black wood (*Picrodendron baccatum*), and canker-berry (*Solanum bahamense*)). In a 1990 follow-up survey of Little Wax Cay, Jordan (pg. 134, 135 in [11]) observed an overall reduction in the weight of individual hutia along with evidence of low rates of reproduction (e.g., few lactating females or palpable testes among males). The low rates of fertility in combination with extensive evidence of hutia over-browsing the plant community led Jordan to conclude that the initial demographic explosion of the Little Wax Cay hutia population was followed by “reproductive depression, probably as a result of nutritional restriction” (pg. 134 in [11]). This population decline occurred in the absence of human presence. Jordan’s findings highlight both the significant impact an introduced hutia population can have on landscapes and food resources, as well as related physiological consequences to hutia reproduction. More broadly, Jordan [11] further reasoned that over time variable plant biodiversity, landscapes, and weather across East Plana Cay, Little Wax Cay, and Warderick Wells will result in heterogonous Bahamian hutia demography and population sizes.

Unlike extant introduced Bahamian hutias on Little Wax and Warderick Wells today, archaeological evidence indicates that the hutias at the Palmetto Junction site would have contended with human presence from the time they were translocated to the island. The abundance of hutia remains at the site suggests that a viable population was successfully established by at least AD 1425. The co-existence of Lucayans and hutias on Providenciales would have necessitated some form of human intervention or management to establish the population and ensure its availability for human consumption. The hutias would have required protection from anthropogenic overexploitation and non-human predation. The introduced hutias also would have needed adequate food resources either naturally available or provisioned by settlers. We suggest that the Lucayans on Providenciales capitalized on the natural adaptive qualities of Bahamian hutias (e.g., broad dietary breadth, efficient water consumption, slow locomotion) in their decision to translocate a population from The Bahamas to the Turks & Caicos Islands. The individuals selected for transport would have been available for capture by hand and tolerant of a boat voyage with limited access to freshwater. Moreover, they would have been able to consume a variety of vascular plants present in the new landscape or food provided directly or indirectly to them through indigenous subsistence practices that may have included cultivated plant resources (e.g., maize). The intentional, human-directed ^13^C-enriched diets for five of the seven sampled hutia individuals serve as a proxy of human agency. Preferentially feeding hutias would have encouraged individual hutia growth, reproduction, and overall survival in a new landscape. Supplementing hutia diet with horticultural crops also would have provided a buffer against possible reductions in hutia size and decreased reproduction as observed on Little Wax Cay due to plant resource depression via persistent hutia browsing over time.

In contrast to modern studies finding that Bahamian hutias are highly vulnerable to human activities and that conservation efforts among extant populations are predicated upon prohibition of all human contact, our findings suggest a complex history of interaction between humans and hutias across the Bahama archipelago. The isotopic and zooarchaeological results from Palmetto Junction suggest that under some conditions Lucayans were able to manage and facilitate viable hutia populations through a combination of translocation, food provisioning, and protection. Despite being consumed as a food resource, indigenous human management and care contributed to the biological success of the introduced hutias at Palmetto Junction.

Corroborating our characterization of hutia management at Palmetto Junction with other zooarchaeological and geochemical evidence remains a challenge (e.g., [12]). First, based on our analysis of strontium ratios across archaeological and modern hutia samples, it is difficult to confidently identify the original geographic provenance of hutias. Hutias have hypsodont molars exhibiting continuous growth and replacement of tooth enamel [81], prohibiting the identification of long-term water and plant consumption that extends beyond local environments. As a result, the identification of translocated hutias in the Bahama archipelago is based on known biogeography and biodiversity across pre- or non-human associated versus human associated contexts. Second, there is a lack of complementary δ^13^C values as well as morphometric data for Bahamian hutias from other sites and islands available for comparison with our results. Therefore, we cannot yet assess whether or not the presumably symbiotic relationship between humans and hutias at Palmetto Junction was characteristic of other islands with Lucayan settlements. It is also possible that the large assemblage of hutias currently reported for only three sites in the archipelago may reflect the of the presence of economic activities among Lucayan archaeological sites, suggesting hutia *management* only occurred at some locations. The relative absence of hutia bones from other Lucayan sites may indicate that hutia *exploitation* was not a significant practice.

Moreover, supplementary archaeological evidence of hutia management, such as pens or corrals for congregating animals or preserved concentrations of dung amassed through captivity, thus far has not been identified. There is also no known archaeological evidence at this time for the extra-culinary use of hutia skins or bones, although they would have been useful materials. Finally, while hutias may have been afforded social importance beyond subsistence, evidence for such practices (e.g., burials of individual animals) is also lacking. Similarly, whether there were specific motivations or goals beyond subsistence for translocating hutias between islands remains unclear. These types of negative evidence are typical across Caribbean sites and highlight the taphonomic challenges in the archaeological study of animal management activities in the Neotropics more generally [21].

## Broader implications and conclusion

Zooarchaeological research shows that Bahamian hutia exposure to anthropogenic impacts extends deep in time to the pre-Columbian era. However, different from extant Bahamian hutia populations under protection from humans today, pre-Columbian populations, such as those represented at Major’s Landing and Palmetto Junction, lived in close proximity to human settlement and horticultural areas. This research demonstrates that by AD 1400 Lucayans successfully translocated and co-existed with Bahamian hutias on Providenciales—suggesting the possibility of a more flexible, symbiotic range of hutia-human interaction in the past than observed in the present. While our interpretation of intentional hutia management cannot be extrapolated to other sites or islands in the Bahama archipelago at this time, our study provides a new baseline and methodological framework for considering the possible scope of human influence over hutia diet, distribution, and population size in the past. Our research holds potential for elucidating the archaeological signatures and consequences of animal translocation and management, and the long-term consequences of these cultural practices among islands in the Caribbean and elsewhere during the Anthropocene.

Identifying the conditions and consequence of indigenous hutia management and consumption through zooarchaeological and isotopic analyses is not a straightforward endeavor. Yet this avenue of research has great potential for how we conceptualize and reconstruct trajectories of past hutia-human interactions informing hutia natural history across the Caribbean, including variable histories of taxonomic distribution and degrees of tolerance to human presence. As demonstrated in our Bahamian hutia case study, the role of past humans in hutia management and biogeography was neither haphazard nor homogenous. Ongoing and future research will benefit from larger-size archaeological samples representing greater spatial, temporal, cultural, and hutia taxonomic diversity, as well as continued morphometric, isotopic, and chronometric analyses, and the integration of ancient DNA analysis. Furthermore, there is great potential for the concerted integration of zooarchaeological datasets within greater Caribbean mammalian biodiversity and conservation research initiatives (e.g., [2]).
